# Based on CRISPR-Cas13a system, to establish a rapid visual detection method for avian influenza viruses

**DOI:** 10.3389/fvets.2023.1272612

**Published:** 2024-01-08

**Authors:** Zongshu Zhang, Chunguang Wang, Xi Chen, Zichuang Zhang, Guoqiang Shi, Xianghe Zhai, Tie Zhang

**Affiliations:** ^1^College of Veterinary Medicine, Hebei Agricultural University, Baoding, China; ^2^Institute of Special Animal and Plant Sciences, Chinese Academy of Agricultural Sciences, Changchun, China; ^3^Hebei Sanshi Biotechnology Co., Ltd., Shijiazhuang, China

**Keywords:** avian influenza virus, Clustered Regularly Interspaced Short Palindromic Repeats, CRISPR associated proteins, recombinase-aided amplification, lateral flow dipstick, nucleic acid detection

## Abstract

To rapidly, specifically, and sensitively detect avian influenza virus (AIV), this research established a visual detection method of recombinase-aided amplification (RAA) based on Clustered Regularly Interspaced Short Palindromic Repeats (CRISPR) and CRISPR associated proteins 13a (Cas13a) system. In this study, specific primers and CRISPR RNA (crRNA) were designed according to the conservative sequence of AIV Nucleprotein (NP) gene. RAA technology was used to amplify the target sequence, and the amplification products were visually detected by lateral flow dipstick (LFD). The specificity, sensitivity, and reproducibility of RAA-CRISPR-Cas13a-LFD were evaluated. At the same time, this method and polymerase chain reaction (PCR)-agarose electrophoresis method were used to detect clinical samples, and the coincidence rate of the two detection methods was calculated. The results showed that the RAA-CRISPR-Cas13a-LFD method could achieve specific amplification of the target gene fragments, and the detection results could be visually observed through the LFD. Meanwhile, there was no cross-reaction with infectious bronchitis virus (IBV), infectious laryngotracheitis virus (ILTV), and Newcastle disease virus (NDV). The sensitivity reached 10^0^ copies/μL, which was 1,000-fold higher than that of PCR-agarose electrophoresis method. The coincidence rate of clinical tests was 98.75 %, and the total reaction time was ~1 h. The RAA-CRISPR-Cas13a-LFD method established in this study had the advantages of rapid, simple, strong specificity, and high sensitivity, which provided a new visual method for AIV detection.

## Introduction

Avian influenza (AI) is an infectious disease caused by avian influenza virus (AIV), belonging to influenza A virus (IAV), which can infect humans. AIV is a negative-strand RNA virus of the orthomyxoviridae family (1, 2), whose genome consists of eight gene segments that encode at least 11 proteins. So far, 18 haemagglutinin (HA) subtypes (H1–H18) and 11 neuraminidase (NA) subtypes (N1–N11) of IAV have been identified (3), among which H5, H7, and H9 subtypes of AIV have a higher incidence in poultry. AI is not only closely related to the economy of poultry industry, but also seriously threatens the health of humans and animals. Once birds were infected, the mortality rate can reach up to 100% (4). Therefore, establishing a rapid and effective detection method for AIV is the key to early and accurate diagnosis of AI. Meanwhile, the visualization of viral nucleic acid detection results makes the primary laboratory and field detection more convenient, which is of great significance for clinical epidemic prevention and control.

Clustered Regularly Interspaced Short Palindromic Repeats (CRISPR) and CRISPR associated proteins (Cas) system is a new discovery in recent years. The CRISPR-Cas system can be used not only in gene editing, but also for nucleic acid detection (5–7). A large number of reporter molecules are added to the reaction system of the CRISPR-Cas system. Under normal circumstances, the reporter molecules will not be cut and remain intact. When the Cas protein and CRISPR RNA (crRNA) form a binary complex to recognize the substrate, the Cas protein is activated and the reporter moleculesare is cut, releasing a large number of signals. At present, to solve the problems of insufficient sensitivity and long time-consuming, the CRISPR-Cas system is combined with nucleic acid amplification technology (8). Recombinase-mediated isothermal nucleic acid amplification is a new type of constant temperature nucleic acid amplification technology, which has the advantages of strong specificity, high sensitivity, short detection time, and easy operation. Under the isothermal condition of 37–42°C, the recombinase binds tightly with the primer DNA to form a complex. Primer recognizes the perfectly matching complementary sequence on the template DNA, with the assistance of single-stranded DNA binding protein and the action of DNA polymerase. Consequently, the double-stranded structure of the template DNA is opened to form a new cDNA strand, and the amplified product grows exponentially (9). Amplification results can be observed by agarose gel electrophoresis, test strips, and real-time fluorescence detection. Based on the CRISPR-Cas system, Zhang Feng's research team (10) combined the CRISPR-Cas13a system with recombinase polymerase amplification (RPA) technology in 2017 to design the specific high-sensitivity enzymatic reporter unlocking (SHERLOCK) detection platform. It has important value in molecular biology diagnosis (6), and has achieved real-time detection in the detection of Ebola virus and Lassa virus (11).

In this study, the CRISPR-Cas13a system, recombinase-aided amplification (RAA), and lateral flow dipstick (LFD) were combined to establish a RAA-CRISPR-Cas13a-LFD method for rapid detection of AIV. The method had the advantages of constant temperature, rapidity, simplicity, specificity, sensitivity, and visualization, which provided a new method for early diagnosis of AI.

## Materials and methods

### Preparation and extraction of nucleic acids

The cDNA of AIV (H5, H7, and H9 subtypes) were stored in our laboratory. RNA of Newcastle disease virus (NDV) and infectious bronchitis virus (IBV) (AV1511) and DNA of infectious laryngotracheitis virus (ILTV) (AV195) were extracted according to the instructions of the viral genome DNA/RNA extraction kit (Tiangen Biochemical Technology Co., Ltd.), in which NDV was a clinically isolated and identified strain preserved in our laboratory, and IBV and ILTV were purchased from the China Institute of Veterinary Drug Control. The RNA of NDV and IBV was reverse transcribed into cDNA using reverse transcription kit and stored for backup.

### Design and synthesis of primers and crRNA

**Figure 1:**
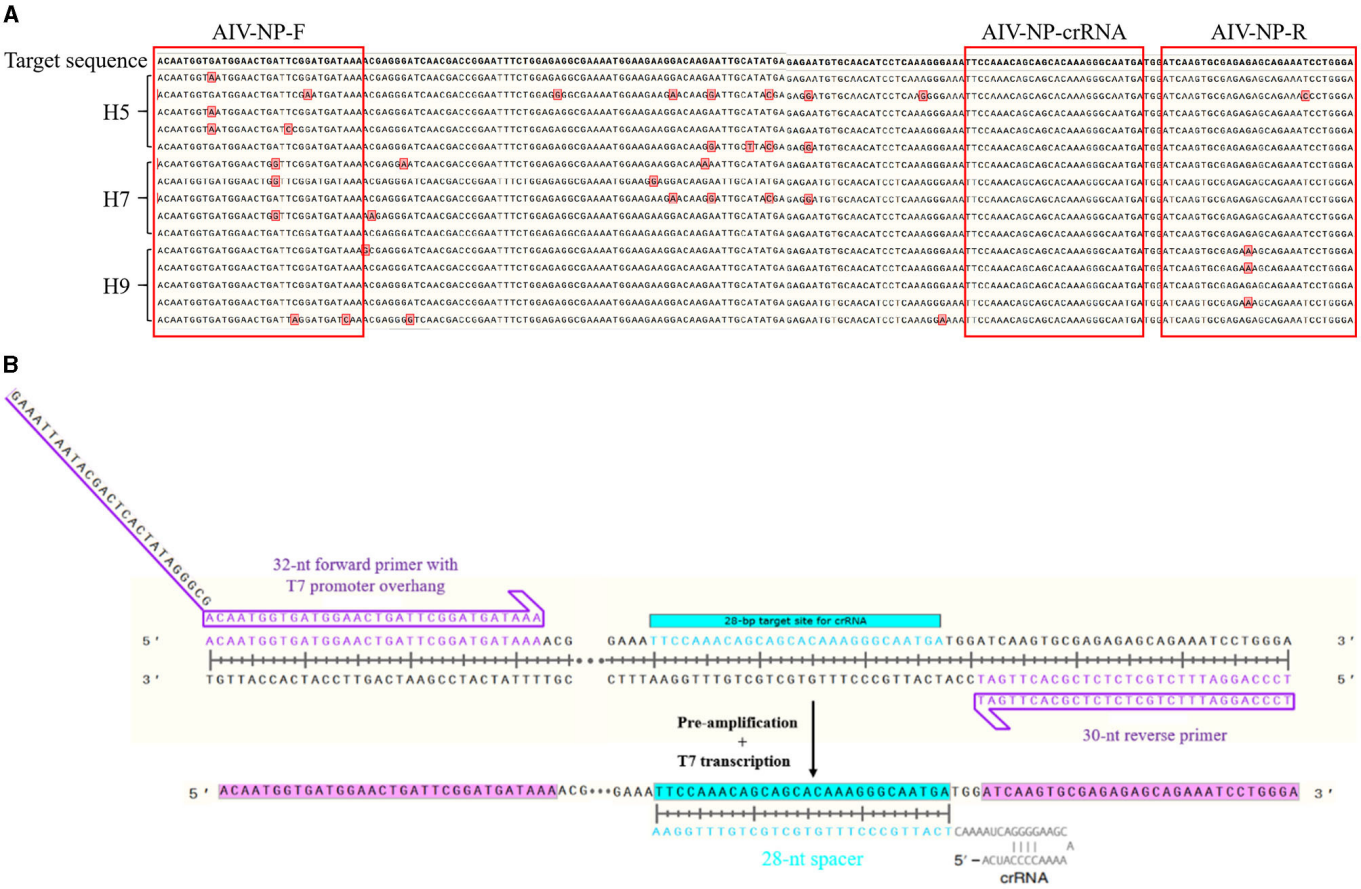
Schematic of primers and crRNA design. **(A)** The alignment results of NP sequences from different subtypes (H5, H7, H9) of AIV; **(B)** Schematic of primers and crRNA design.

More than 100 NP gene sequences of AIV were searched from NCBI, which were analyzed and compared by DNAMAN. The relatively conserved region of Nucleprotein (NP) gene (GenBank: MW265408.1) was selected to design the upstream and downstream primers and crRNA. Figure 1A showed the results of NP sequence comparison of some H5, H7, and H9 subtypes of AIV. Since Cas13a detection was involved, a T7 promoter has to be added at the 5′ end of its upstream primer to allow T7 transcription, while a crRNA complementary to the target sequence was designed (Figure 1B). The primers, crRNA *in vitro* transcription template, and T7-3G oligonucleotides (Table 1) were synthesized by Sangon Bioengineering Co., Ltd. (Shanghai, China).

**Table 1:** Primer and crRNA sequences utilized in the study.

**Name**	**Sequence (5^′^ → 3^′^)**	**Location (bp)**
AIV-NP-F	GAAATTAATACGACTCACTATAGGGACAATGGTGATGGAACTGATTCGGATGATAAA	562–593
AIV-NP-R	TCCCAGGATTTCTGCTCTCTCGCACTTGAT	719–748
AIV–NP-crRNA	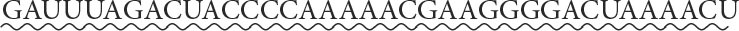 UCAUUGCCCUUUGUGCUGCU GUUUGGAA	688–715
AIV-crRNA-IVT	TTCCAAACAGCAGCACAAAGGGCAATGA 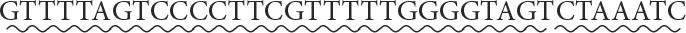 CCCTATAGTGAGTCGTATTAATTTC	688–715
T7-3G oligonucleotide	GAAATTAATACGACTCACTATAGGG	—
AIV-NP-PCR-F	ACAATGGTGATGGAACTGATTC	562–583
AIV-NP-PCR-R	TCCCAGGATTTCTGCTCTCTC	728–748

_, T7 promoter sequence; ~, neck ring structure.

### Preparation and purification of crRNA

Mixed 10 μL reaction system:1 μL crRNA-IVT (100 μM), 1 μL T7-3G oligonucleotide (100 μM), 1 μL Standard Taq buffer (10 × ), 7 μL UltraPure water, and then denature it at 95°C for 5 min in a polymerase chain reaction (PCR) instrument, subsequently, slowly refrigerate it to 4°C at 0.1°C/s. After the reaction, 10 μL of the reaction product was mixed with 10 μL of NTP buffer mix (New England Biolabs, cat.no. E2050S), 2 μL T7 RNA polymerase mix (New England Biolabs, cat.no. E2050S), 17 μL UltraPure water and then transcribed overnight at 37°C. Subsequently, slowly refrigerate it to 4°C at 0.1°C/s. After the reaction, the products were purified by using the Spin Column RNA Cleanup & Concentration Kit (Sangon Bioengineering Co., Ltd., Shanghai, China). Before store it in the refrigerator at −80°C for backup, the concentration was measured.

### Establishment of RAA-CRISPR-Cas13a-LFD reaction system

The reaction system of 50 μL was prepared according to the instructions of the nucleic acid test strip quick detection kit (Anhui Microanaly Genetech Co., Ltd., Hefei, China): Buffer A 25 μL, nucleic acid-free water 14 μL, upstream primer (10 μM) 2 μL, downstream primer (10 μM) 2 μL, nucleic acid extract 4 μL, MgOAC 3 μL, mixed the above reagents and reacted at 39°C for 30 min. After the reaction, 1 μL amplification product was mixed with 8 μL detection solution D, and 1 μL crRNA (30 ng/μL) was added, and the reaction was placed at 37°C for 30 min.

After the above amplification reaction was completed, 40 μL diluent L was added to 10 μL reactant and thoroughly mixed. The LFD was directly inserted into the mixture, and the results could be directly observed within 5–10 min. The result of detection is positive if both the C line and T line appear (The C line is located in the quality control area, T line is located in the detection area), and negative if only the C line appears.

### Specificity evaluation of RAA-CRISPR-Cas13a-LFD for AIV detection

The cDNA of AIV (H5, H7, and H9 subtypes), NDV, IBV, and DNA of ILTV were used as templates, and ddH_2_O was set as the negative control to perform the RAA-CRISPR-Cas13a-LFD to evaluate the specificity of the reaction.

### Construction of standard plasmids

AIV (H9 subtype) cDNA was used as the template for PCR amplification. A 50 μL reaction system included 25 μL 2 × Taq Mix, 1 μL each of upstream and downstream primers (10 μM), 2 μL template, and 21 μL ddH_2_O. The reaction procedure was as follows: pre-denaturation at 94°C for 5 min, 35 cycles of 94°C for 30 s, 53°C for 30 s, and 72°C for 30 s, and then the plates were extended at 72°C for 5 min. The PCR products were subjected to 2% agarose gel electrophoresis. After gel recovery, the T-Vector pMD 20 (Takara Biomedical Technology Co., Ltd., Beijing, China) was ligated and introduced into DH5α competent cells, which were coated and cultured on LB medium containing ampicillin resistance for blue-white screening to extract the plasmids. The number of DNA copies per unit volume of plasmid was calculated according to Moore's law. The calculation formula is:

Plasmid copy number (copies/μL) = [plasmid concentration (g/μL) × 6.02 × 10^23^]/{[Vector length (bp) + fragment length (bp)] × 660 g/mol}

The constructed plasmids were diluted to 10^9^-10^0^ copies/μL by 10-fold dilution method and stored at −20°C for backup.

### Sensitivity evaluation of RAA-CRISPR-Cas13a-LFD for AIV detection

The sensitivity was evaluated by RAA-CRISPR-Cas13a-LFD method, PCR-agarose electrophoresis method, and quantitative polymerase chain reaction (qPCR) method, respectively, using the diluted plasmids as templates and setting ddH_2_O as a negative control.

The RAA-CRISPR-Cas13a-LFD method: 10^5^-10^0^ copies/μL of plasmid was used as the template, and the reaction was performed according to the established system and procedure.

The PCR-agarose electrophoresis method: 10^7^-10^0^ copies/μL of plasmid was used as the template, and the reaction was performed according to the reaction system and procedure of plasmid construction.

The qPCR method: 25 μL reaction system included TB Green Premix Ex Taq II (TliRNaseH Plus) (2 × ) (Takara Biomedical Technology Co., Ltd., Beijing, China) 12.5 μL, each of PCR upstream and downstream primers (10 μM) 1.0 μL, template (10^6^-10^0^ copies/μL plasmid) 1.0 μL and ddH_2_O 9.5 μL. The reaction procedure was pre-denaturation at 95°C for 30 s; denaturation at 95°C for 5 s, annealing at 55°C for 30 s, and extension at 72°C for 30 s, and the results were observed after 40 cycles.

### Reproducibility and stability evaluation of RAA-CRISPR-Cas13a-LFD for AIV detection

10^5^, 10^3^, and 10^1^ copies/μL plasmids were selected as templates, and each concentration was repeated three times with a negative control to evaluate the reproducibility and stability of RAA-CRISPR-cas13a-LFD.

### Validation of assay performance on clinical samples

PCR-agarose electrophoresis method and RAA-CRISPR-Cas13a-LFD method detected 160 throat swab samples from diseased chickens (the samples were from 8 chicken farms in North China), which were initially diagnosed as AI according to clinical symptoms, respectively. The PCR-agarose electrophoresis method was used as a standard to compare the coincidence rate of the two methods.

## Results

### Analytical specificity of RAA-CRISPR-Cas13a-LFD method

**Figure 2:**
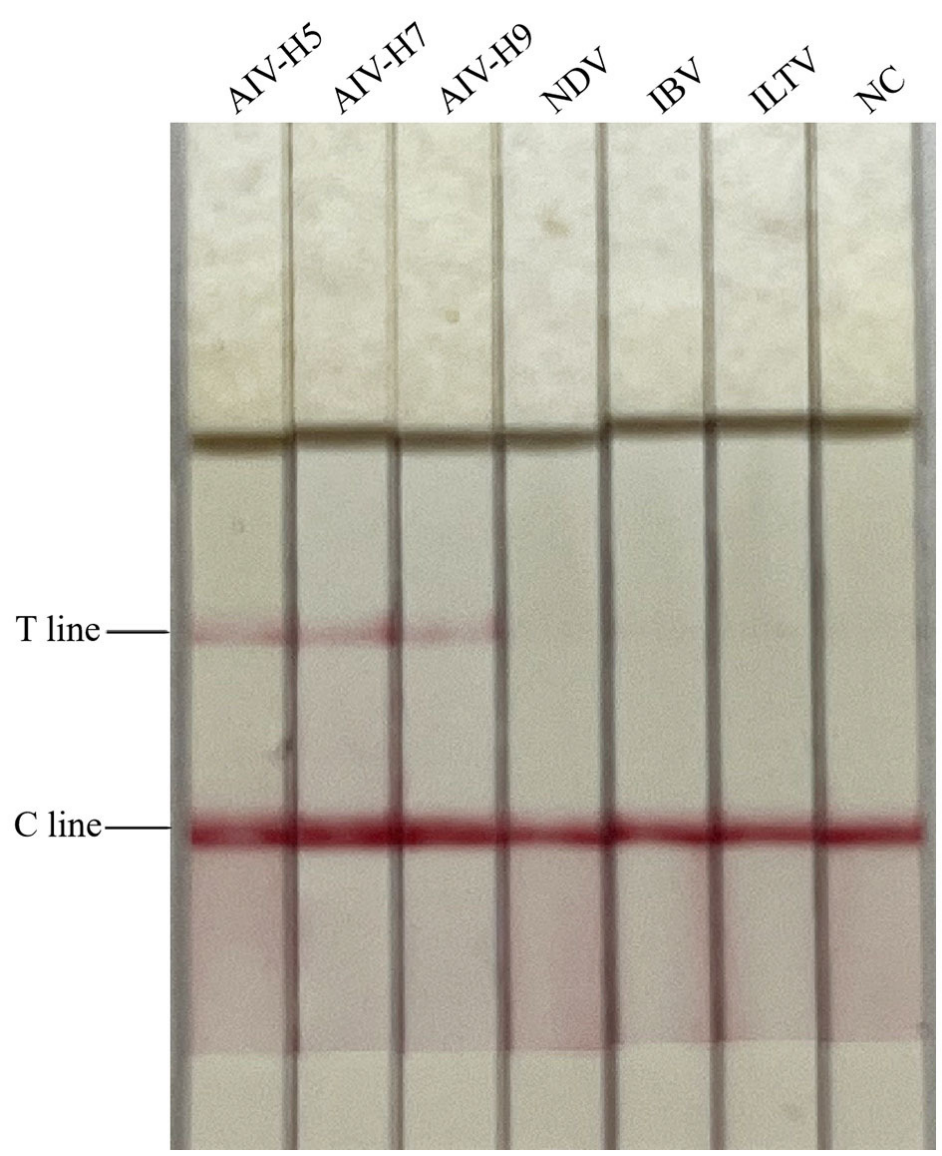
Specificity test results of RAA-CRISPR-Cas13a-LFD method for AIV detection. NC, negative control. Only AIV-H5, AIV-H7, and AIV-H9 showed T and C lines, while other viruses and negative control only showed C line.

The cDNA of AIV (H5, H7, and H9 subtypes), NDV, and IBV, and the DNA of ILTV were, respectively, used as templates for detection, and the results were shown in Figure 2. Only AIV (H5, H7, and H9 subtypes) showed double bands of T and C lines, while NDV, IBV, and ILTV showed only C lines. It showed that only AIV was detected positively, indicating that the RAA-CRISPR-Cas13a-LFD method established in this study had good specificity.

### Analytical sensitivity of RAA-CRISPR-Cas13a-LFD method

**Figure 3:**
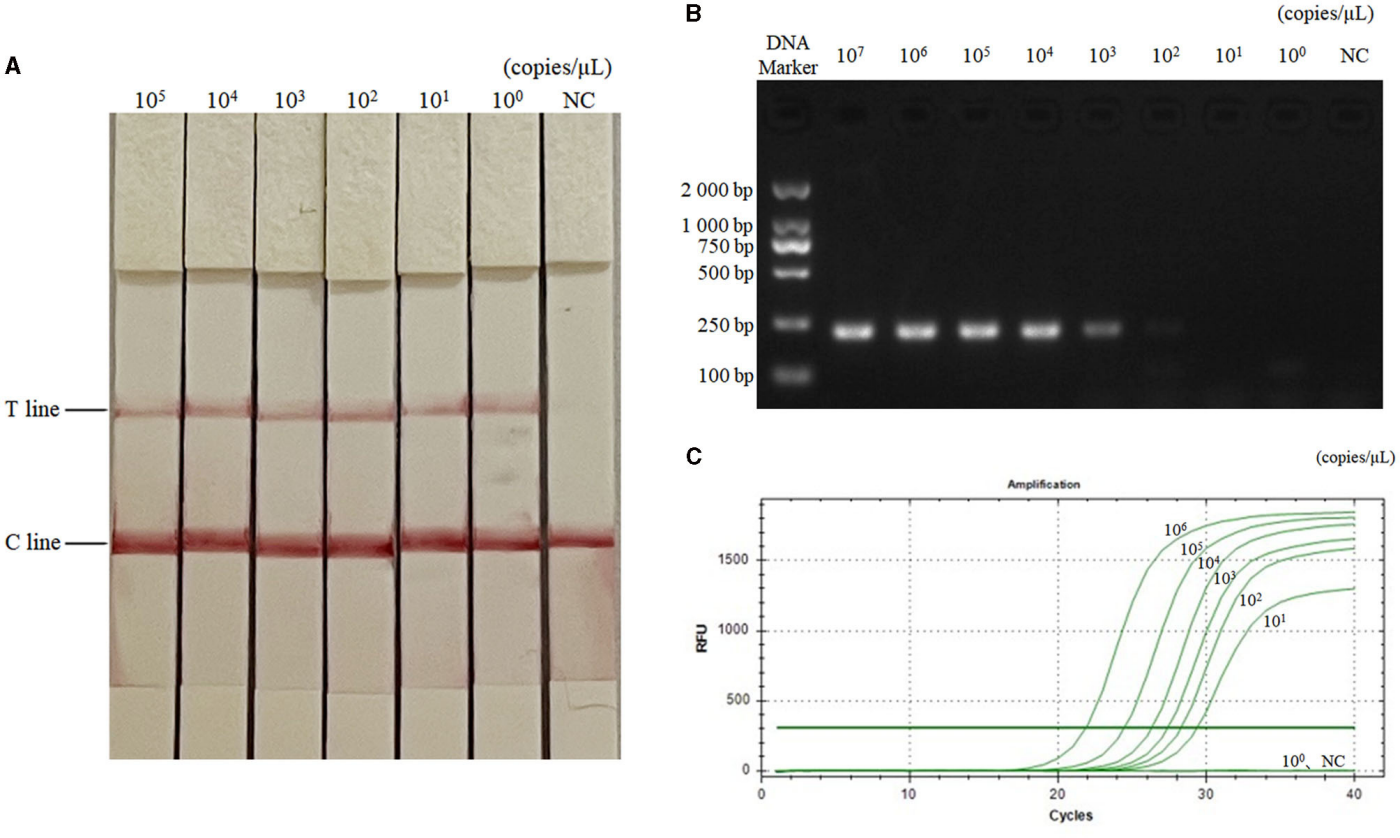
Sensitivity test results of the three methods for AIV detection. **(A–C)** Were the results of AIV detection by RAA-CRISPR-Cas13a-LFD method, PCR-agarose electrophoresis method, and qPCR method, respectively. NC, negative control. In **(A)**, the lowest detection limit of RAA-CRISPR-Cas13a-LFD method was 10^0^ copies/μL. In **(B)**, the lowest detection limit of PCR-agarose electrophoresis method was 10^3^ copies/μL. In **(C)**, the lowest detection limit of qPCR was 10^1^ copies/μL.

The diluted plasmids were used as templates, and the sensitivity was tested by RAA-CRISPR-Cas13a-LFD method, PCR-agarose electrophoresis method, and qPCR method, respectively. Results were shown in Figure 3, the RAA-CRISPR-Cas13a-LFD method could still show clear T lines at the template concentration of 10^0^ copies/μL, and its lowest detection limit could reach 10^0^ copies/μL. The lowest detection limit of PCR-agarose electrophoresis and qPCR was 10^3^ and 10^1^ copies/μL, respectively. It could be demonstrated that the RAA-CRISPR-Cas13a-LFD method established in this study had high sensitivity.

### Analytical reproducibility and stability of RAA-CRISPR-Cas13a-LFD method

**Figure 4:**
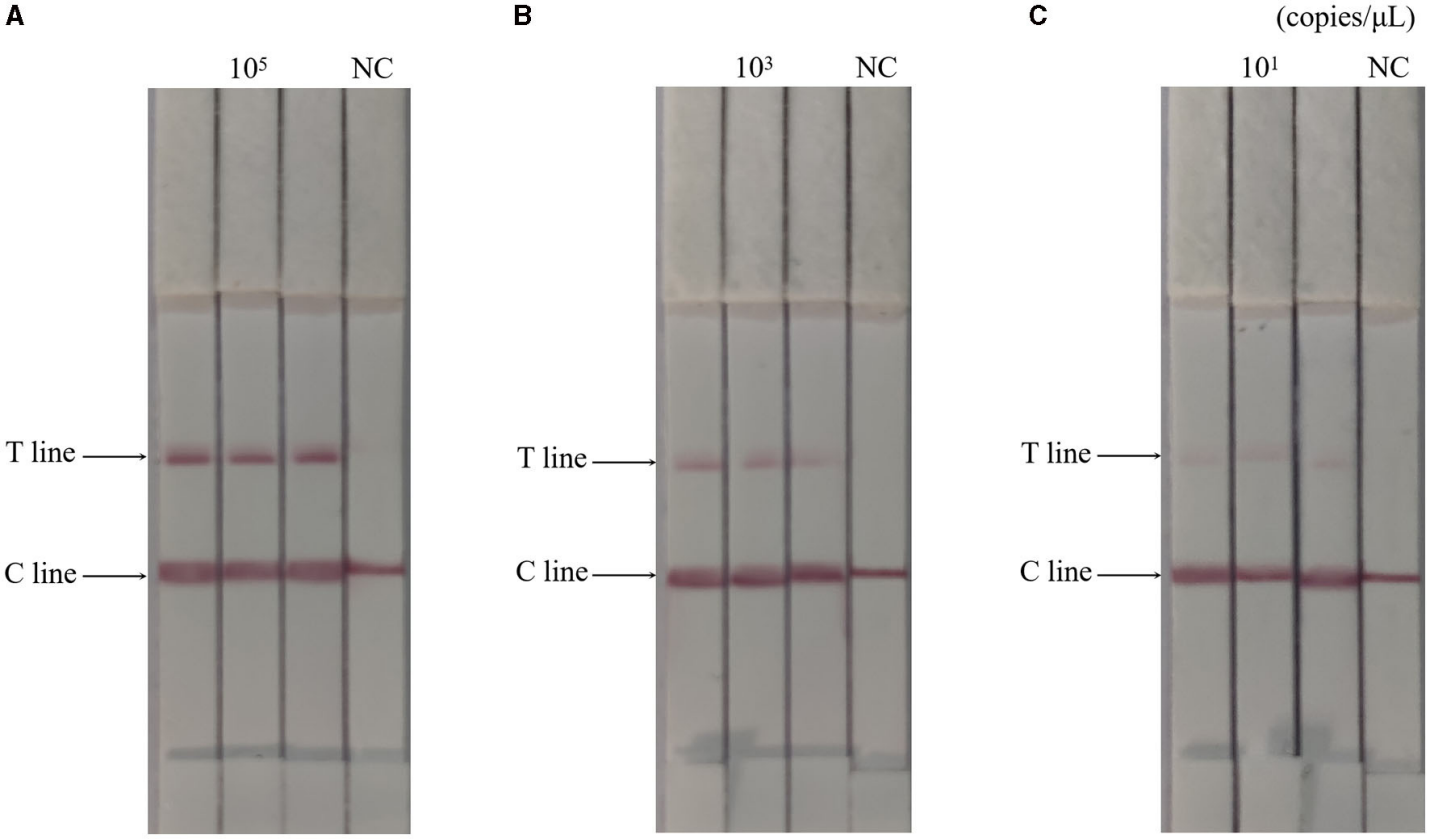
Reproducibility test results of RAA-CRISPR-Cas13a-LFD method for AIV detection. **(A–C)** Were the results of three replicate tests at template concentrations of 10^5^, 10^3^, and 10^1^ copies/μL, respectively. NC, negative control. The T and C lines appeared in all three replicate groups, and only the C line appeared in the negative control.

The RAA-CRISPR-Cas13a-LFD method was used to perform three replicate tests on 10^5^, 10^3^, and 10^1^ copies/μL of AIV plasmids, respectively, while negative controls were set up. Results were shown in Figure 4, the results of the AIV sample detection group were all positive, while the results of the control group were all negative, indicating that the RAA-CRISPR-Cas13a-LFD method established in this study had good reproducibility and stability.

### Analytical of assay performance on clinical samples

A total of 160 throat swab samples of clinically suspected diseased chickens with AI were collected from 8 chicken farms in North China. After extracting nucleic acid, the samples were detected by the RAA-CRISPR-Cas13a-LFD method established in this study and PCR-agarose electrophoresis method, respectively. The results were shown in Table 2, and the coincidence rate of the two methods was 98.75%, indicating that the RAA-CRISPR-Cas13a-LFD method could be applied for clinical field detection of AIV.

**Table 2:** Clinical test results of the two methods.

**Detection method**	**Positive (copy)**	**Negative (copy)**	**Positive rate (%)**	**Coincidence rate (%)**
PCR-agarose electrophoresis	35	125	21.88	98.75
RAA-CRISPR-Cas13a-LFD	37	123	23.13	

Coincidence rate (%) = (number of all positive samples + number of all negative samples in the two test methods compared)/total number of samples × 100 %.

## Discussion

AI is the number one killer in poultry farming, and highly pathogenic avian influenza (HPAI) can cause mass mortality in the event of an outbreak. Some studies have shown that some low pathogenic avian influenza (LPAI) can be transformed into HPAI. Meanwhile, as a zoonotic disease, AI seriously threatens public safety and human health (12).

Currently, there are various detection methods for AIV, the main detection methods are pathogenic and serological tests. Among the pathogenic detection methods, molecular biological detection methods are commonly used, and PCR and qPCR are two representative methods. However, they require expensive thermal cycling equipment and professional operators, which are not conducive to clinical field use. Serological tests include haemagglutination test, hemato-inhibition test, enzyme-linked immunosorbent assay, and AGAR gel diffusion test, etc. However, these methods are not suitable for clinical promotion due to their non-specific errors and false-positive results.

With the development of science and technology, isothermal nucleic acid amplification techniques have emerged, such as loop-mediated isothermal amplification (LAMP), nucleic acid sequence-based amplification (NASBA), rolling circle amplification (RCA), RAA, RPA, etc. The above isothermal amplification methods have greatly improved the specificity and sensitivity of detection, but they also have shortcomings. The design of LAMP primer is very complex, which requires special design software, and improper operation is prone to false positives, thus affecting the accuracy of results (13). NASBA can only use RNA as a template when combining with primers, which has limitations and requires three enzymes to amplify together, resulting in high cost and consumption. RCA has a relatively long reaction time and relatively poor selectivity of the linking enzymes, resulting in low sensitivity. RAA/RPA is a new isothermal rapid nucleic acid amplification technology, which uses recombinase, single-stranded binding protein, and DNA polymerase to amplify under isothermal conditions (14). Due to its advantages of isothermal amplification and short reaction time, it is now widely applied.

In recent years, the discovery of CRISPR-Cas system has revolutionized biology, enabling accurate editing of almost any DNA or RNA molecule (15–18). Some Cas proteins have been found to have accessory cleavage activity, such as Cas12a and Cas13a. Cas12a binds crRNA to form ribonucleo protein, which cleaves single-stranded DNA and double-stranded DNA targets containing crRNA complementary sequences, has weak side branch activity, and makes nucleic acid detection less sensitive (16). For targeting recognition and cleavage, it is limited by the PAM site, which has less selectivity when detecting short sequences and is difficult to meet the requirements of effectors (6). Cas13a enzyme does not require a strict sequence preference at the target site, allowing it to select a broader range of sequences for detection (10, 19). Of course, CRISPR-Cas13a may exhibit non-specific effects where non-target RNA sequences similar to the target sequence can interfere with the recognition of CRISPR-Cas13a, leading to unnecessary cleavage. However, in this experiment, the T7 promoter sequence was introduced during the pre-amplification process, and the amplified products were transcribed into abundant RNA using T7 RNA polymerase. Activated by crRNA guidance, Cas13a exhibits collateral activity and cuts specific RNA targets (14, 20), thereby avoiding non-specific recognition by CRISPR-Cas13a and ensuring the sensitivity and specificity of the reaction.

This study combined CRISPR-Cas13a, RAA, and LFD to establish a rapid, specific, and sensitive method for the visual detection of AIV. First of all, the nature of crRNA with different targeting makes RAA-CRISPR-Cas13a-LFD method with good specificity, no cross-reactivity with IBV, ILTV, NDV. In the detection of AIV, and the time consumed is half of that of PCR agarose electrophoresis method. Secondly, a T7 promoter sequence was added to the 5′ end of the upstream primer for RAA amplification. The application of T7 RNA polymerase in the detection reaction greatly improved the amplification efficiency, and significantly improved the sensitivity of the detection, which was 1,000-fold higher than that of the PCR-agarose electrophoresis method, and single-copy detection was achieved. Meanwhile, compared with the RT-RAA-LFD method for AIV (the lowest detection limit is 10^2^ copies/μL) previously established by our research team (21), it is more sensitive. Chang et al. (22) used CRISPR-Cas13a to detect porcine reproductive and respiratory syndrome virus, and the detection limit was 172 copies/μL, which was slightly lower than the sensitivity of this study. Yang et al. (23) established a multiplex real-time RT-PCR method for detecting H5, H7, and H9 subtype AIV, and the detection limit was 1–10 copies (plasmid DNA) per reaction, but it required sophisticated reaction equipment. Thirdly, reproducibility examination of the RAA-CRISPR-Cas13a-LFD method showed reproducible results with good stability. Fourthly, the results of the RAA-CRISPR-Cas13a-LFD method were displayed by the LFD to realize the visualization of the detection results. Finally, in the application of testing clinical samples, two samples showed different results, so we performed repeat testing and sequencing analysis on these two samples. The results showed that these two samples did contain AIV. Based on the comprehensive analysis, two possible reasons were obtained, one was the low virus content of these two samples, and the other was the higher sensitivity of RAA-CRISPR-Cas13a-LFD method established in this study compared to the PCR-agarose electrophoresis method. In conclusion, the RAA-CRISPR-Cas13a-LFD method established in this study has the advantages of convenience, rapidity, high specificity, high sensitivity, and visualization of test results. It can be applied to primary clinical tests and some areas lack laboratory resources. It provides a new technical means for clinical and field detection of AIV.

## Data availability statement

The original contributions presented in the study are included in the article/supplementary material, further inquiries can be directed to the corresponding author.

## Author contributions

ZoZ: Conceptualization, Data curation, Formal analysis, Methodology, Writing – original draft. CW: Writing – review & editing. XC: Investigation, Writing – review & editing. ZiZ: Writing – review & editing. GS: Investigation, Writing – review & editing. XZ: Methodology, Supervision, Writing – review & editing. TZ: Conceptualization, Methodology, Project administration, Supervision, Writing – review & editing.
